# Cell-Free DNA as a New Biomarker of IVF Success, Independent of Any Infertility Factor, Including Endometriosis

**DOI:** 10.3390/diagnostics13020208

**Published:** 2023-01-05

**Authors:** Maria Manuel Casteleiro Alves, Luísa Oliani, Micaela Almeida, Henrique José Cardoso, António Hélio Oliani, Luiza Breitenfeld, Ana Cristina Ramalhinho

**Affiliations:** 1 Health Sciences Research Centre (CICS), Faculty of Health Sciences, University of Beira Interior (UBI), 6201-506 Covilhã, Portugal; 2 Assisted Reproduction Laboratory of Academic Hospital of Cova da Beira (CHUCB), 6200-251 Covilhã, Portugal; 3 School of Sciences, University of Minho, 4710-057 Braga, Portugal; 4 Department of Obstetrics and Gynecology, São José do Rio Preto School of Medicine (FAMERP), São José do Rio Preto 15090-000, Brazil

**Keywords:** cfDNA, follicular fluid, embryo quality, endometriosis, PCOS, POF, infertility, IVF success

## Abstract

Cell-free DNA fragments detected in blood and in other biological fluids are released from apoptotic/necrotic cells. In this study, we analyzed cfDNA levels in follicular fluid (FF) samples from patients with infertility. Samples were collected from 178 infertile women and cfDNA was extracted and quantified by qPCR, using ALU115 and ALU247 primers, and statistical correlations were performed. We found that cfDNA concentration was significantly higher in FF pools from women aged 35 and over than in women under 35 years of age (*p* = 0.017). We also found that q247 cfDNA levels were significantly higher in women with an associated female factor, such as endometriosis, PCOS and POF, compared with women with no specific cause of infertility (*p* = 0.033). The concentration of cfDNA did not vary significantly in each group of women with an associated female factor. The concentration of cfDNA was significantly higher in the FF of women that obtained embryos with a high fragmentation rate, compared to embryos with a low fragmentation rate (*p* = 0.007). Finally, we found that women who did not become pregnant during IVF treatments had higher q247 cfDNA levels (*p* = 0.043). The quantification of cfDNA could be an important biomarker of follicular micro-environment quality to predict embryo quality and the success of IVF, making them more specific and effective.

## 1. Introduction

Infertility is a disease characterized by the failure to establish a clinical pregnancy after 12 months of regular, unprotected sexual intercourse or due to an impairment of a person’s capacity to reproduce either as an individual or with his/her partner. Worldwide, about 1 in 10 couples develop primary or secondary infertility. Fertility interventions may be initiated in less than one year based on medical, sexual and reproductive history, age, physical findings and diagnostic testing. We often associate infertility with a female cause; however, infertility is a disease of the couple which may have female, male or mixed causes [1]. Several disorders are known causes for female infertility: namely, polycystic ovary syndrome (PCOS), endometriosis and premature ovarian failure (POF). In addition, tubal factors and ovulatory events and disorders can contribute to infertility. Up to a third of couples are diagnosed with unexplained infertility, or infertility of an unknown cause. Unexplained infertility abnormalities are likely to be present but cannot be detected by current methods. Several disorders are known causes for male infertility such as oligozoospermia; asthenozoospermia; azoospermia; teratozoospermia; and varicocele [2]. Cryptic sperm defects in apparently normal spermatozoa may be the cause of total fertilization failure in oocyte donor cycles [3]. Thus, including the gender dimension in the diagnostics and therapeutics of infertile couples helps to eliminate gender bias, indicating how to design equitable access to infertility diagnosis and treatment [4].

In in-vitro fertilization (IVF) treatments, the selection of embryos with greater potential for implantation is limited, depending mainly on morphological criteria. Embryonic classification via embryo morphology is very subjective, despite being the most used nowadays. Some of these limitations include comparing, grouping and statistically stratifying embryos to determine the most viable embryo for implantation [5,6]. It is therefore necessary to identify new biomarkers that are more accurate, allowing for the adaptation of treatments to each woman, making them more specific and effective.

Several studies have shown that the follicular fluid (FF), which surrounds the oocyte, is directly involved in follicular maturation, oocyte growth and development [7,8,9]. In addition, it can be easily collected, being aspirated along with oocytes during the IVF process and its composition is an important indicator of oocyte and embryo quality and may represent a safe source of new biomarkers that will enable the identification and standardization of a more effective and non-invasive method of oocyte quality evaluation [10,11] and could be used in supplementary prognostic/diagnostic tools in IVF treatments [10,12].

Cell-free DNA (cfDNA) is characterized by non-cell-bound, double-stranded DNA fragments present in human plasma or serum [13]. The discovery of cfDNA in plasma brought many innovations in several areas of medicine, making them a marker with growing interest due to their clinical applications, expanding the possibilities of non-invasive diagnosis and prognosis [6,14]. Biological fluids such as peripheral blood, cerebrospinal fluid, urine, saliva and follicular fluid, among others, can be used in liquid biopsy for the detection and isolation of cfDNA [14,15]. How cfDNA is released into the bloodstream is still poorly understood. Studies show that cellular events such as necrosis, apoptosis and secretion by the cells themselves determine the number of cfDNA fragments, which can be released through an active or passive mechanism [16]. Nuclear and mitochondrial DNA can be passively released into the blood from apoptotic or necrotic cells [17] and then phagocytosed by macrophages in healthy individuals, keeping the basal cfDNA level low [18]. Circulating DNA can also be actively released by cells, leading to a significant increase in cfDNA levels, as observed in some cancers and other serious pathologies [19]. Cells that undergo programmed cell death (apoptosis) are accompanied by a physiologically orchestrated deterioration of genomic DNA, which results in small fragments (180–200 base pairs). On the other hand, in cell necrosis, a large variety of fragments with different sizes are formed due to random digestion [20].

Previous studies that analyzed fetal circulating DNA in maternal plasma were essential for the development of non-invasive prenatal diagnostic tools [21]. These fetal DNA tests are used to analyze the most common trissomies and numerical alterations of the sex chromosomes. Thus, cfDNA from circulating cells is considered a potential biomarker of female infertility [22,23]. There are few studies linking cfDNA concentrations in follicular fluid with infertility factors. On what concerns endometriosis, a study correlated elevated levels of cfDNA in plasma with endometriosis when compared to controls [24]. Regarding polycystic ovary syndrome (PCOS), a study reported that cfDNA levels are significantly higher in small follicles compared to large ones [16]. There are some studies that aim to relate cfDNA levels in follicular fluid with the success rates of IVF and with embryonic development. Czamanski-Cohen et al. (2014) reported that increased plasma cfDNA levels were associated with low pregnancy rates in women undergoing IVF treatments. Later, the same group showed that the stress caused by the IVF process was responsible for the increase of cfDNA in women’s blood; apparently, relaxation techniques helped to significantly reduce the levels of cfDNA in blood, improving the results of IVF treatments [25]. Scalici et al. (2014) conducted a study on the detection and quantification of cfDNA in follicular fluid to evaluate if it could be used for the development of an innovative prognostic test for the assessment of embryonic quality. The level and integrity of cfDNA with the size of the follicle were studied, along with the clinical characteristics of women undergoing conventional IVF or ICSI, and the results of IVF treatment. They found that high levels of cfDNA in FF samples were correlated with poor embryo quality on the third day of implantation, suggesting that cfDNA quantification in both follicular fluid and serum could give better prediction of embryo quality and could be used routinely beyond subjective morphological criteria. Traver et al. (2015) investigated whether cfDNA levels in follicular fluid samples from women undergoing IVF treatments could be related to their ovarian reserve status, controlled ovarian stimulation (COS) protocols and the results of IVF treatments. It was observed that the cfDNA level was significantly higher in FF samples from women with low ovarian reserve than in women with normal ovarian reserve. The cfDNA levels were also significantly higher in women who received long-term ovarian stimulation or high doses of gonadotropins. The cfDNA levels were also shown to have significant predictive value for pregnancy outcomes, with 88% specificity and 60% sensitivity [26].

In summary, cfDNA quantification is believed to be a non-invasive biomarker that could help to determine the chance of IVF success in the future. This technique could help patients who have had multiple in vitro fertilization failures, and who have already tried other specific techniques without success [27]. As mentioned above, there are some studies that relate the quantification of cfDNA with infertility; however, as far as we are aware, there are none with a larger sample size than ours. Furthermore, this is the only study performed in infertile women that performed a statistical comparison of cfDNA for each ALU primer (q115 and q247), in addition to the q247/q115 ratio. Therefore, this study was designed to complement the existing studies; thus, we analyzed cfDNA levels in FF samples from patients with infertility.

## 2. Materials and Methods

### 2.1. Study Population

**Figure 1:**
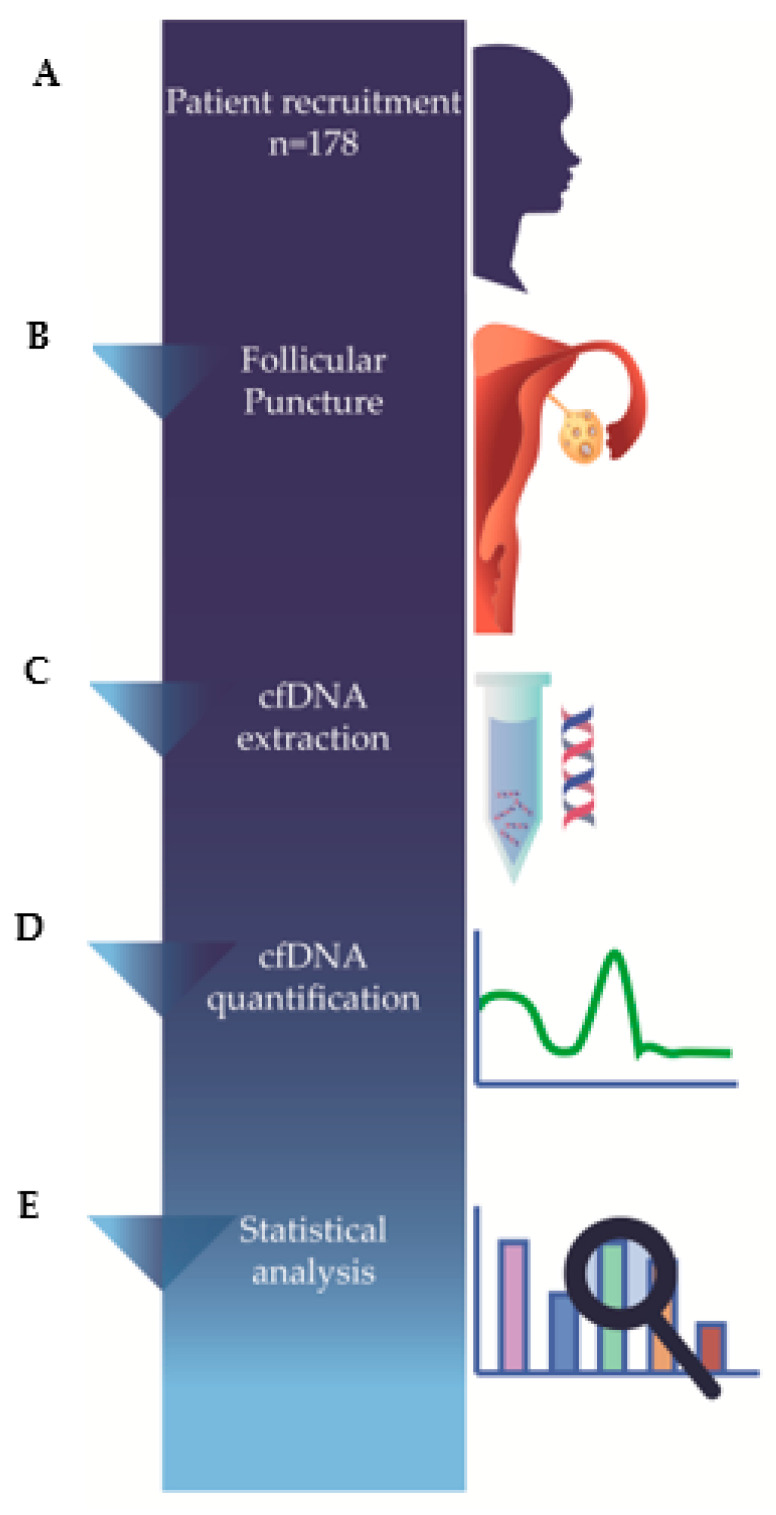
Schematic summarization of methods. A total of 178 FF samples were collected (**A**); oocyte retrieval was performed by follicular puncture by transvaginal ultrasound-guided aspiration, 36 h after the injection of human chorionic gonadotrophin (**B**); samples were centrifuged, supernatants were filtered and digested with PK, followed by PK heat inactivation and insolubilization (**C**); cfDNA was quantified by qPCR, using ALU115 and ALU247 primers (**D**); statistical tests were performed using Statistical Package for the Social Sciences (SPSS^®^) for Windows (version 24), IBM Corp., Amonk, NY, USA and results were considered significant when *p* ≤ 0.05 (**E**).

In total, we collected 178 FF samples of women under 39 years of age that failed to establish a clinical pregnancy after 12 months of regular, unprotected sexual intercourse, followed at the Assisted Reproduction Unit from the Academic Hospital Center of Cova da Beira, Covilhã, Portugal. A total of 45 women had endometriosis, 60 were diagnosed with PCOS, 40 presented premature ovarian failure (POF) and 44 women were considered as controls with no specific cause of female infertility. Women were enrolled between October 2015 and July 2019 (Figure 1A). The inclusion criteria were: women under 39 years of age that failed to establish a clinical pregnancy after 12 months of regular, unprotected sexual intercourse, followed at the Assisted Reproduction Unit from Academic Hospital Center of Cova da Beira, Covilhã, Portugal, that were submitted to conventional IVF or ICSI as a treatment to achieve pregnancy, that signed the informed consent form. The exclusion criteria were: infertile women above 39 years, or that did not require conventional IVF or ICSI as infertility treatment, and women that met inclusion criteria but did not sign informed consent.

### 2.2. Ethical Approval

The study was approved by the Ethical Committee of the Academic Hospital Center of Cova da Beira, Covilhã, Portugal (reference number 47/2015, approved on 15 July 2015). Informed consent was obtained from cases before participating in the study. 

### 2.3. Follicular Fluid Sample Collection

Oocyte retrieval was performed by transvaginal ultrasound-guided aspiration 36 h after the injection of human chorionic gonadotrophin and each follicle was aspirated. To avoid any blood contamination, only clear fluid samples were included, whereas bloodstain and cloudy follicular fluid samples were excluded (Figure 1B).

### 2.4. Follicular Fluid Preparation

All FF samples from the same patient were pooled and a volume of 15 mL was centrifuged at 3000× *g* for 15 min. Supernatants were filtered with 0.2 μm filters to eliminate cell debris and then stored −80 °C until cfDNA extraction. A total of 178 FF pools were collected for this study.

### 2.5. Cell-Free DNA Extraction

FF pools were prepared for cfDNA quantification as previously reported [28]. Specifically, 20 μL of each FF pool was digested with 16 μg proteinase K (PK) (Qiagen Sciences, Germantown, MD, USA) in 20 μL of buffer (25 mL/L Tween 20, 50 mmol/L Tris and 1 mmol/L EDTA) at 50 °C for 20 min, followed by PK heat inactivation and insolubilization at 95 °C for 5 min. After centrifugation at 10,000× *g* for 5 min, supernatants were removed and stored at −80 °C for cfDNA quantification (Figure 1C).

### 2.6. Cell-Free DNA Quantification

The cfDNA was quantified by real-time polymerase chain reaction (qPCR), using ALU115 and ALU247 primers. The ALU115 primer amplified both short (apoptotic) and long (non-apoptotic) DNA fragments, whereas the ALU247 primer amplified long non-apoptotic DNA fragments only. The ALU-qPCR result obtained with ALU115 primers represents the total amount of serum DNA. This allows the calculation of DNA integrity by using the q247/q115 ratio, which represents the proportion of cfDNA generated by necrosis over total cfDNA [28]. The sequences of the primers were as follows: ALU115 forward, 5′-CCTGAGGTCAGGAGTTCGAG-3′ and reverse, 5′-CCCGAGTAGCTGGGATTACA-3′; ALU247 forward, 5′-GTGGCTCACGCCTGTAATC-3′ and reverse, 5′-CAGGCTGGAGTGCAGTGG-3′. Each ALU-qPCR reaction included 1 μL of PK-digested FF pool and a reaction mixture containing 0.25 μm of forward and reverse ALU115 or ALU247 primers and 10 μL of Maxima SYBR Green qPCR Master Mix (2×) (Thermo Scientific, Waltham, MA, USA). ALU-qPCR reaction conditions were optimized to yield optimal results to suit the equipment, reagents and conditions of our laboratory. Standard curves were created for both (ALU115 and ALU247 primers) sets by PCR amplifying serially diluted human genomic DNA samples. A negative control (without template) was integrated in each qPCR plate and each FF pool was analyzed in duplicate (Figure 1D).

### 2.7. Statistical Analysis

Univariate analysis was performed for each variable. Continuous parametric data are presented as mean ± standard deviation (SD) and categorical variables as numbers and percentages. Analysis of variance (ANOVA) or a Mann–Whitney test was used to compare cfDNA levels based on the homogeneity of variances by the Levene test and normality of the distribution assessed using the Shapiro–Wilk test. A multiple linear regression (GLM) as multivariable analysis was performed for the variables infertility factor, βhCG analysis result, age and embryo fragmentation. Statistical tests were performed using SPSS^®^. Results were considered significant when *p* ≤ 0.05 (Figure 1E).

## 3. Results

This prospective study recruited 178 women enrolled in conventional IVF and ICSI. The clinical and pathological characteristics are shown in Table 1. The mean age was 34 ± 3.69 years (range: 19 to 39 years) and body index mass (BMI) was 24 ± 3.70 kg/m^2^ (range: 17 to 33 kg/m^2^). The mean age at menarche was 12 years ± 1.82 (range: 8 to 18 years) and the infertility length was 53 months (range: 14 to 192 months). 

The calculation of DNA integrity by using the q247/q115 ratio represents the proportion of cfDNA generated by necrosis over total cfDNA. The mean of q247/q115 ratio was 0.99 (SD: 0.12) in follicular fluid samples (n = 178), suggesting that the cfDNA analyzed was mainly originated from cellular necrotic events. 

We performed statistical comparison for each ALU primer (q115 and q247) separately, in addition to the ratio of q247/q115 (cfDNA concentration) for all parameters. Table 1 analyses age, BMI, ethnicity, age at menarche, smoke habits and infertility length. Regarding age, we found that cfDNA concentration was significantly higher in FF pools from women aged 35 and over than in women under 35 years of age (*p* = 0.017). Concerning BMI, 123 women presented less or equal 25 kg/m^2^ BMI and 55 had more than 25 kg/m^2^ BMI. FF cfDNA concentration was not significantly different between these two groups. Considering ethnicity, 174 women were Caucasian, 2 were Gypsy and 2 were Afro-Europeans. We did not find statistically significant differences between these groups. On what concerns age at menarche, 102 women had their first menstruation before 13 years, 52 between 13 and 14 years and 21 presented an age at menarche over 15 years. FF cfDNA concentration was not significantly higher in either group. Regarding smoking habits, 130 women never smoked, 21 previously smoked and in 27 smoking habits are present. When we compared these groups, in terms of cfDNA concentration, we did not find statistically significant differences. Considering infertility length, 9 women presented infertility for less than 24 months, 91 had an infertility time between 24 and 48 months and 78 had been infertile for more than 48 months. FF cfDNA concentration was not significantly different between the three groups. 

Female factors and women with no specific cause of infertility are presented in Table 2. Regarding the female infertility factor, 45 women had endometriosis, 60 were diagnosed with PCOS, 40 presented POF and 44 women were considered as controls with no specific cause of female infertility. FF cfDNA concentration did not vary significantly when we compared each group of women with an associated female factor (endometriosis, PCOS or POF) with the other female factors. However, q247 cfDNA levels were significantly higher in women with any female factor, when compared with women with no specific cause of infertility (idiopathic) (*p* = 0.033).

AMH, AFC, days of stimulation and total dose of gonadotropins are described in Table 3. Concerning anti-Müllerian hormone (AMH) level, 35 women had low AMH serum concentration at day 3 of the menstrual cycle (≤1 ng/mL) and 139 had normal AMH (greater than 1 ng/mL). We verified that FF cfDNA concentration did not vary significantly between women with low AMH serum concentration and women with normal AMH. Concerning antral follicle count (AFC), 110 women had low count of AFC (≤10) and 62 had a normal count of AFC (>10). FF cfDNA concentration was not significantly different between samples of women with low AFC and samples of women with normal AFC. Regarding days of stimulation, 99 women had a long ovarian stimulation (>10 days) and 78 had a short treatment (≤10 days). We did not find statistically significant differences between long ovarian stimulation and short treatment. Considering the dose of gonadotropins, 113 women received a high total dose of gonadotropins (>3000 IU/I) and 64 women were treated with a lower dose (<3000 IU/I). There was no statistically significant difference between women who received a high total dose of gonadotropins and women stimulated with a lower dose.

Table 4 analyzes retrieved oocytes, mature oocytes and total embryo number. Considering the number of retrieved oocytes, 37 women had less or equal than 6 retrieved oocytes and 140 had more than 6 retrieved oocytes. We did not find statistically significant differences between these two groups. Regarding mature oocytes, 101 women had less than 8 mature oocytes (MII), 35 had between 8 to 12 MII and 40 had more than 12 MII. When we compared the number of MII we did not find a statistically significant difference between the groups. Concerning the number of embryos cleaved in day 3 post-fertilization, 47 women had a small number of embryos (≤2) and 131 obtained more than 3 embryos. We did not find a statistically significant difference between the obtainment of fewer than 2 embryos compared to more than 3 embryos obtained.

Table 5 includes the percentage of embryo fragmentation and beta chorionic gonadotropin hormone (βhCG) analysis result. In view of embryo fragmentation percentage, 81 embryos presented a high fragmentation rate (≥20%) and 37 embryos had a low fragmentation rate (<20%). We found a statistically significant difference between these two groups, as cfDNA concentration was significantly higher in FF pools of women that obtained embryos with a high fragmentation rate than in FF pools of women that obtained embryos with a low fragmentation rate (*p* = 0.007). Lastly, 44 women had a positive βhCG result (pregnant women) and 84 had a negative result (non-pregnant women). When we compared the results of pregnant women with non-pregnant women, we found that women who did not become pregnant during IVF treatments had higher q247 cfDNA levels than women who had a confirmed pregnancy (*p* = 0.043).

Finally, we performed a multiple linear regression to verify whether the variables infertility factor, βhCG analysis result, age and embryo fragmentation can predict cfDNA concentration values. The analysis resulted in a statistically significant model [F = 4, 112) = 2.531; *p* = 0.044; R^2^ = 0.083]. However, in this model, only embryo fragmentation was considered as a predictor of cfDNA concentration values (β = 0.250; t = 2.579; *p* = 0.011). 

## 4. Discussion

First, it is important to note that our study calculated the DNA integrity by using the q247/q115 ratio, which represents the proportion of cfDNA generated by necrosis over total cfDNA. The q247/q115 ratio mean value in the 178 FF samples was 0.99 (SD: 0.12), suggesting that cfDNA mainly originated from cellular necrotic events. Thus, cfDNA in FF samples in our population was primarily the outcome of necrotic processes and, as far as we are aware, none of the other studies where cfDNA was quantified in follicular fluid obtained a similar result. It is also important to point out that none of the other studies carried out on cfDNA quantification in follicular fluid, to our knowledge, analyzed a number of samples as large as we did. As previously stated, DNA fragments are the result of apoptotic or necrotic events and can be easily detected in blood and other body fluids [29,30] including FF [15]. The cfDNA level is reported to be increased in some cancers and other severe diseases and is already used as a non-invasive biomarker for their early detection and/or prognosis [17,22,31]. The major source of cfDNA in healthy individuals is apoptotic cells; therefore, a preponderance of longer DNA fragments has been proposed as a marker for disorder/disease detection [32].

This study demonstrates that the cfDNA concentration was significantly higher in FF pools from women aged 35 and over than in women under 35 years of age. A previous study reported that blood cfDNA level was higher in IVF patients suffering from stress [25]. Moreover, it has been shown that relaxation techniques may be beneficial during the IVF process, to reduce plasma cfDNA levels and to improve pregnancy outcomes [25]. Therefore, a long period of stress caused by the absence of pregnancy could lead to an increase in apoptotic and necrotic events in follicular cells and ultimately to higher FF cfDNA levels [26]. The level of stress may explain the increase in cfDNA concentration in older women, maybe because they are often aware of the greater risks associated with pregnancies at an older age and the known association between increasing age and decreasing success in pregnancy rate. This set of factors can increase the stress level and consequently the level of cfDNA in follicular fluid and have repercussions on the IVF success rate. Therefore, it is important that the level of stress and the evolutionary implications of parental competence on the development of children born by IVF are monitored, as described in the study carried out by Sofia Burgio [33]. 

We also found that q247 cfDNA levels were significantly higher in women with an associated female factor than in women with no specific cause of infertility. In our study, we divided our sample according to three infertility factors: endometriosis, PCOS and POF. These factors have similar cellular and/or molecular backgrounds. They may be originated by the imbalance caused between reactive oxygen species (ROS) and antioxidants. Increasing evidence suggests that oxidative stress may act as an important risk factor for reproductive disorders. Habib et al. suggested that the management of endometriosis requires a holistic approach focused on reducing overall inflammation, increasing detoxification, and attenuating troublesome symptoms [34]; several studies have put the attention on the role of oxidative stress, which may be implicated in the pathophysiology of endometriosis, causing a general inflammatory response in the peritoneal cavity [35]. Elevated levels of cfDNA in plasma were detected in patients diagnosed with endometriosis compared with controls [24]. This increase in plasmatic cfDNA could be caused by apoptotic events within the endometriosis tissue. ROS overproduction was also described to be causative in POF progression [36,37]. ROS have a damaging effect on DNA integrity in the developing oocyte and are responsible for a high copy number of intra-follicular DNA [38], so it is reasonable to believe that cfDNA levels are higher when these pathologies are present. In relation to PCOS, a study previously reported that PCOS is associated with follicular maturity abnormalities, such as an increased number of small pre-antral follicles [39,40]. Furthermore, another study reported that cfDNA levels are significantly higher in small follicles compared to larger ones [15]. These small follicles could contain high cfDNA levels, thus explaining why cfDNA concentration is high in patients with PCOS. Additionally, to optimize ovarian response in women with poor ovarian reserve, at oocyte retrieval day, the physician is more meticulous in the aspiration of smaller follicles, to try to increase the number of retrieved oocytes. Thus, follicular fluids from smaller follicles would become proportionally more represented in PCOS patients than in women who respond normally to the stimulus [26]. These data corroborate our results, which demonstrate that women who have an associated infertility factor have a higher level of cfDNA than those who do not have any associated infertility factor.

We also verified that cfDNA concentration was significantly higher in FF pools of embryos with a high fragmentation rate than with a low fragmentation rate. Our study agrees with others previously carried out. Scacili et al. (2014) demonstrated that the cfDNA level in follicular fluid samples corresponding to top-quality embryos was significantly lower than in follicular fluid samples related to poor-quality embryos. Traver et al. (2015) also found that the cfDNA level in follicular fluid samples was strongly and significantly associated with embryo quality at Day 3. Additionally, a study detected mitochondrial and genomic DNA in spent embryo culture medium and found that the mitochondrial cfDNA concentration was higher in medium samples in which bad-quality cleavage embryos were cultured compared with medium from top-grade embryos [41]. In clinical practice, and although there are already some methods that help to select the best quality embryo, it is not always easy to select the best embryo, and doubts are often created as to which transferred embryo will result in pregnancy. Cell-free DNA quantification could give a better prediction of embryo quality and could be used routinely beyond subjective morphological criteria.

**Figure 2:**
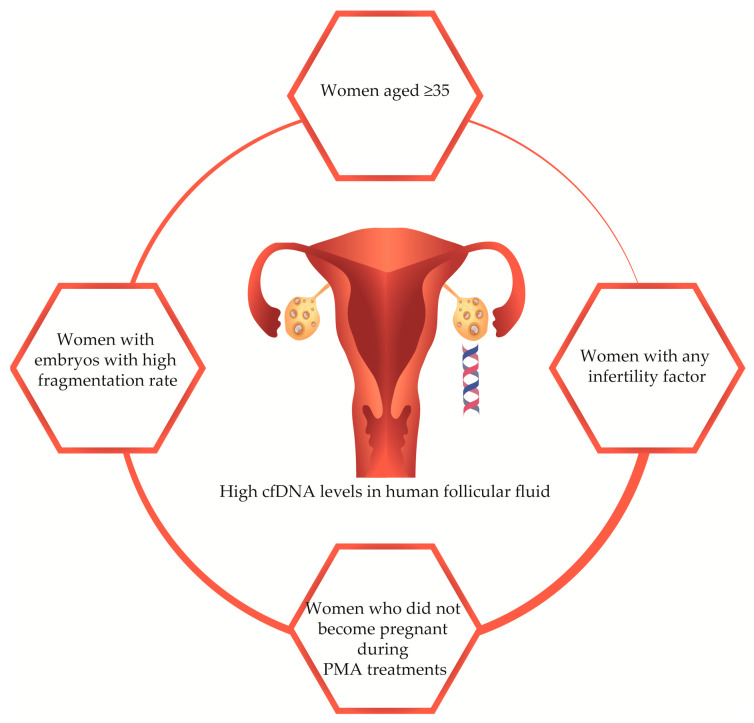
Schematic model of the influence of high cfDNA levels in human follicular fluid, according to our results. Age above 35 years seems to be associated with higher cfDNA concentration; in women with any female infertility factor, cfDNA levels are higher, if compared with women with no specific cause of infertility; cfDNA concentration is higher in women that obtained poor quality embryos; cfDNA levels are higher in women who had no success in pregnancy achievement after IVF treatment.

We found that women who did not become pregnant after IVF treatments had higher q247 cfDNA levels than women who had a confirmed pregnancy. Czamanski-Cohen et al. (2014) reported that increased plasmatic cfDNA was associated with low pregnancy rates in women submitted to the IVF process. In the same study, they also reported that maternal cell apoptotic or necrotic events led to an increase in circulating cfDNA levels leading to a hostile environment for conception [25]. Traver et al. (2015) showed that FF cfDNA level was an independent and significant predictive factor for pregnancy outcome, with 88% specificity and 60% sensitivity. All these results add up and lead us to conclude that cfDNA quantification can be a great facilitator to predict higher success rates in IVF (Figure 2).

Finally, we performed a multiple linear regression to verify whether the variables infertility factor, βhCG analysis result, age and embryo fragmentation can predict cfDNA concentration values. To perform this regression, six presuppositions must be met, namely: the value of n must be greater than 20 for each independent variable; residual values must be independent; residuals must be normally distributed; homoscedasticity, absence of multicollinearity and absence of outliers. Our data meet all presuppositions. The analysis resulted in a statistically significant model [F = 4, 112) = 2.531; *p* = 0.044; R^2^ = 0.083]. In this model, only embryo fragmentation was considered as a predictor of cfDNA concentration values (β = 0.250; t = 2.579; *p* = 0.011). This result enforces our previous results and allows us to state that this predictive model could be used as a supplemental tool for determining the chance of IVF success. 

A limitation of our study is the use of exogenous gonadotropins in short and long PCOS protocols to increase the number of retrieved oocytes, because the use of exogenous gonadotropins interferes with follicular steroidogenesis at certain levels and might be one of the causes of higher concentration of cfDNA in FF. So, further research considering IVF populations during a non-stimulated menstrual cycle is required. It is also very important to include a control group in the study and use more primers to determine the apoptotic or necrotic origin of cfDNA. In the future, we also aim to evaluate cfDNA concentration in the serum of stimulated women, because another limitation of this study was not having performed real-time PCR in the serum of these women. In addition, future studies that include genetic sequencing of DNA fragments to determine the origin of the cfDNA would be helpful to recognize potential pathologies that affect fertility and prevent successful implantation after IVF treatments.

In conclusion, this study indicates that cfDNA quantification is a non-invasive biomarker, can help to choose top-quality embryos to transfer and can be an aid when determining the chance of IVF success independently of the infertility cause. The identification of new biomarkers that have greater precision in the assessment of embryonic quality can contribute to an improvement in infertility treatments personalization and, therefore, to an increase in the success of IVF, where the assessment of cfDNA concentration in follicular fluid samples can then function as a non-invasive biomarker of embryo quality. It is extremely important to consolidate the optimization of the different existing procedures to make cfDNA analysis routine during the IVF process. Research in this area should continue, as this new test could be incorporated into the program of current practice and improve personalized patient care. Cell-free DNA shows great promise as a new biomarker; however, there are still many questions that are difficult to overcome. Even though it is considered an easy and quick technique to perform, it is also very costly. So, it is fundamental that the cost of analysis becomes more reasonable.

## Figures and Tables

**Table 1:** Cell-free DNA levels according to age, BMI, ethnicity, age at menarche, smoking habits and infertility length.

Variable	Mean	*n*	Min-Max	SD	FF cfDNA (ng/uL) Mean ± SD [95%CI] (q115)	*p*-Value (q115)	FF cfDNA (ng/uL) Mean ± SD [95%CI] (q247)	*p*-Value (q247)	FF cfDNA (ng/uL) Mean ± SD [95%CI] (q247/q115)	*p*-Value (q247/q115)
Age (years)	34	-	19–39	3.69	-	-	-	-	-	-
<35 years	-	92	-	-	1.3 ± 1.7 [1.0–1.7]	0.259	1.7 ± 2.9 [1.1–2.3]	0.670	9.7 ± 1.1 [9.5–9.9]	0.017
≥35 years	-	86	-	-	1.5 ± 1.8 [1.1–1.9]		1.3 ± 1.8 [0.9–1.7]		10.1 ± 1.2 [9.9–10.4]	
BMI (kg/m^2^)	24	-	17–33	3.70	-	-	-	-	-	-
≤25 kg/m^2^	-	123	-	-	1.5 ± 1.7 [1.2–1.8]	0.612	1.6 ± 2.7 [1.1–2.1]	0.427	9.9 ± 1.2 [9.7–10.2]	0.582
>25 kg/m^2^	-	55	-	-	1.4 ± 1.6 [0.9–1.8]		1.3 ± 1.7 [0.8–1.7]		9.8 ± 1.2 [9.5–10.2]	
Ethnicity	-	-	-	-	-	-	-	-	-	-
Caucasian	-	174	-	-	1.4 ± 1.7 [1.1–1.8]	0.922	1.5 ± 2.4 [1.1–1.8]	0.743	9.9 ± 1.1 [9.7–10.1]	0.065
Gypsy	-	2	-	-	0.9 ± 1.2 [1.0–1.2]		2.2 ± 2.5 [2.0–2.5]		9.1 ± 0.7 [2.7–15.6]	
Afro-Europeans	-	2	-	-	1.3 ± 0.9 [0.7–9.9]		0.4 ± 0.4 [0.3–4.3]		11.7 ± 2.4 [10.2–33.8]	
Age at menarche (years)	12	-	8–18	1.82	-	-	-	-	-	-
≤12 years	-	102	-	-	1.4 ± 1.6 [1.0–1.7]	0.675	1.7 ± 2.8 [1.1–2.2]	0.403	9.8 ± 1.2 [9.6–10.1]	0.348
13–14 years	-	52	-	-	1.4 ± 1.7 [0.9–1.9]		1.1 ± 1.3 [0.7–1.5]		10.0 ± 1.2 [9.7–10.4]	
≥15 years	-	21	-	-	1.7 ± 2.2 [0.7–2.7]		1.4 ± 2.3 [0.4–2.5]		10.2 ± 1.1 [9.7–10.7]	
Smoking Habits	-	-	-	-	-	-	-	-	-	-
Never	-	130	-	-	1.4 ± 1.7 [1.1–1.7]	0.998	1.5 ± 2.5 [1.0–1.9]	0.741	10.0 ± 1.2 [9.7–10.2]	0.572
Previous	-	21	-	-	1.4 ± 1.5 [0.7–2.1]		1.7 ± 2.6 [0.5–2.9]		9.9 ± 1.2 [9.4–10.4]	
Present	-	27	-	-	1.4 ± 1.7 [1.1–1.7]		1.2 ± 1.5 [0.6–1.8]		9.7 ± 1.7 [9.2–10.2]	
Infertility length (months)	53	-	14–192	32.8	-	-	-	-	-	-
<24 months	-	9	-	-	0.6 ± 0.8 [0.3–1.2]	0.277	0.5 ± 0.3 [0.3–0.8]	0.476	9.7 ± 1.02 [8.9–10.5]	0.435
24–48 months	-	91	-	-	1.5 ± 1.8 [1.1–1.9]		1.6 ± 2.3 [1.1–2.1]		9.8 ± 1.2 [9.6–10.1]	
>48 months	-	78	-	-	1.3 ± 1.6 [1.0–1.7]		1.5 ± 2.4 [0.9–2.1]		10.0 ± 1.1 [9.8–10.3]	

SD, standard deviation; BMI, body mass index. Missing data from 3 women in age at menarche.

**Table 2:** Cell-free DNA levels according to infertility factor.

Variable	*n*	FF cfDNA (ng/uL) Mean ± SD [95%CI] (q115)	*p*-Value (q115)	FF cfDNA (ng/uL) Mean ± SD [95%CI] (q247)	*p*-Value (q247)	FF cfDNA (ng/uL) Mean ± SD [95%CI] (q247/q115)	*p*-Value (q247/q115)
Endometriosis	45	1.2 ± 1.4 [0.8–1.6]	0.528	1.3 ± 1.5 [0.8–1.7]	0.376	9.9 ± 1.3 [9.5–10.3]	0.822
PCOS	60	1.2 ± 1.5 [0.8–1.6]	0.359	1.6 ± 3.5 [0.7–2.5]	0.566	10.0 ± 1.2 [9.6–10.3]	0.599
POF	40	1.6 ± 1.9 [1.0–2.2]	0.494	1.5 ± 2.1 [0.8–2.2]	0.999	10.0 ± 1.01 [9.7–10.4]	0.326
Idiopathic	44	1.8 ± 2.1 [1.1–2.4]	0.187	1.6 ± 1.5 [1.1–2.0]	0.033	9.8 ± 1.1 [9.4–10.1]	0.375

PCOS, polycystic ovary syndrome; POF, premature ovarian failure.

**Table 3:** Cell-free DNA levels according to AMH, AFC, days of stimulation and total dose of gonadotropins.

Variable	Mean	*n*	Min-Max	SD	FF cfDNA (ng/uL) Mean ± SD [95%CI] (q115)	*p*-Value (q115)	FF cfDNA (ng/uL) Mean ± SD [95%CI] (q247)	*p*-Value (q247)	FF cfDNA (ng/uL) Mean ± SD [95%CI] (q247/q115)	*p*-Value (q247/q115)
AMH (ng/mL)	3.40	-	0.02–27	3.64	-	-	-	-	-	-
≤1 ng/mL	-	35	-	-	1.6 ± 1.9 [0.9–2.3]	0.479	1.7 ± 2.2 [0.9–2.5]	0.534	9.9 ± 1.2 [9.5–10.3]	0.940
>1 ng/mL	-	139	-	-	1.4 ± 1.7 [1.1–1.7]		1.4 ± 2.5 [1.0–1.9]		9.9 ± 1.2 [9.7–10.1]	
AFC	9.98	-	3–27	4.51	-	-	-	-	-	-
≤10	-	110	-	-	1.4 ± 1.7 [1.0–1.7]	0.533	1.6 ± 2.3 [1.2–2.1]	0.466	9.8 ± 1.1 [9.6–10.1]	0.300
>10	-	62	-	-	1.5 ± 1.8 [1.1–2.0]		1.3 ± 2.7 [0.6–2.0]		10.0 ± 1.2 [9.7–10.4]	
Days of stimulation	10.7	-	8–14	1.18	-	-	-	-	-	-
≤10	-	99	-	-	1.3 ± 1.8 [0.9–1.6]	0.251	1.2 ± 2.0 [0.8–1.6]	0.070	9.9 ± 1.1 [9.7–10.2]	0.483
>10	-	78	-	-	1.6 ± 1.7 [1.2–2.0]		1.9 ± 2.8 [1.2–2.5]		9.9 ± 1.3 [9.6–10.2]	
Total dose of gonadotropins (IU/I)	2383	-	150–3900	783.7	-	-	-	-	-	-
<3000 IU/I	-	113	-	-	1.5 ± 1.8 [1.2–1.9]	0.317	1.5 ± 1.8 [1.2–1.9]	0.625	9.9 ± 1.2 [9.7–10.1]	0.838
≥3000 IU/I	-	64	-	-	1.4 ± 1.8 [0.9–1.8]		1.2 ± 1.6 [0.8–1.6]		9.9 ± 1.1 [9.7–10.2]	

SD, standard deviation; AMH, anti-Müllerian hormone; AFC, antral follicle count. Missing data from 4 women in AMH variable; 6 women in AFC variable; 1 woman in days of stimulation; and 1 woman in total dose of gonadotropins.

**Table 4:** Cell-free DNA levels according to retrieved oocytes, mature oocytes and total embryo number.

Variable	Mean	*n*	Min-Max	SD	FF cfDNA (ng/uL) Mean ± SD [95%CI] (q115)	*p*-Value (q115)	FF cfDNA (ng/uL) Mean ± SD [95%CI] (q247)	*p*-Value (q247)	FF cfDNA (ng/uL) Mean ± SD [95%CI] (q247/q115)	*p*-Value (q247/q115)
Retrieved oocytes	9	-	0–32	5.85	-	-	-	-	-	-
≤6	-	37	-	-	1.7 ± 2.0 [1.0–2.4]	0.268	1.7 ± 2.3 [0.9–2.5]	0.614	10.0 ± 1.3 [9.5–10.4]	0.661
>6	-	140	-	-	1.3 ± 1.6 [1.1–1.7]		1.4 ± 2.4 [1.0–1.8]		9.9 ± 1.2 [9.7–10.1]	
Mature oocytes	8	-	0–27	5.41	-	-	-	-	-	-
<8	-	101	-	-	1.5 ± 1.7 [1.1–1.8]	0.315	1.4 ± 1.8 [1.1–1.8]	0.848	9.9 ± 1.1 [9.7–10.1]	0.378
8–12	-	35	-	-	1.7 ± 2.1 [1.0–2.5]		1.1 ± 0.9 [0.7–1.4]		10.1 ± 1.1 [9.7–10.5]	
≥13	-	40	-	-	0.9 ± 1.1 [0.6–1.3]		2.0 ± 4.1 [0.7–3.3]		9.7 ± 1.3 [9.3–10.2]	
Total embryo number	5	-	0–20	4.03	-	-	-	-	-	-
≤2	-	47	-	-	1.7 ± 1.8 [1.1–1.9]	0.257	1.5 ± 2.1 [0.9–2.2]	0.832	10.1 ± 1.2 [9.7–10.5]	0.210
>2	-	131	-	-	1.3 ± 1.7 [1.0–1.6]		1.5 ± 2.5 [1.0–1.9]		9.8 ± 1.1 [9.6–10.1]	

SD, standard deviation. Missing data from 1 woman in retrieved oocytes variable and 2 women in mature oocytes variable.

**Table 5:** Cell-free DNA levels according to embryo fragmentation and pregnancy result.

Variable	*n*	FF cfDNA (ng/uL) Mean ± SD [95%CI] (q115)	*p*-Value (q115)	FF cfDNA (ng/uL) Mean ± SD [95%CI] (q247)	*p*-Value (q247)	FF cfDNA (ng/uL) Mean ± SD [95%CI] (q247/q115)	*p*-Value (q247/q115)
% embryo fragmentation	-	-	-	-	-	-	-
% fragmentation < 20	37	1.1 ± 1.3 [0.6–1.5]	0.107	1.4 ± 1.3 [0.9–1.9]	0.937	9.5 ± 1.0 [9.2–9.9]	0.007
% fragmentation ≥ 20	81	1.6 ± 1.8 [1.2–2.0]		1.5 ± 2.6 [0.9–2.0]		10.2 ± 1.2 [9.9–10.4]	
βhCG analysis result	-	-	-	-	-	-	-
Positive	44	1.6 ± 1.7 [1.1–2.1]	0.146	1.3 ± 1.4 [0.9–1.8]	0.043	9.9 ± 1.0 [9.5–10.2]	0.377
Negative	84	1.3 ± 1.7 [1.0–1.7]		1.4 ± 2.6 [0.8–2.0]		10.1 ± 1.2 [9.8–10.4]	

βhCG, beta chorionic gonadotropin hormone. Missing data from 60 women in embryo fragmentation variable and 50 women in pregnancy result variable.

## Data Availability

Not applicable.
